# Copper-Modified Titania-Based Photocatalysts for the Efficient Hydrogen Production under UV and Visible Light from Aqueous Solutions of Glycerol

**DOI:** 10.3390/nano12183106

**Published:** 2022-09-07

**Authors:** Anna Yu. Kurenkova, Anastasiya Yu. Yakovleva, Andrey A. Saraev, Evgeny Yu. Gerasimov, Ekaterina A. Kozlova, Vasily V. Kaichev

**Affiliations:** 1 Federal Research Center, Boreskov Institute of Catalysis SB RAS, Lavrentieva Ave. 5, 630090 Novosibirsk, Russia; 2 Department of Natural Science, Novosibirsk State University, Pirogova St. 1, 630090 Novosibirsk, Russia

**Keywords:** photocatalysis, hydrogen evolution, titanium dioxide, anatase, rutile

## Abstract

In this study, we have proposed titania-based photocatalysts modified with copper compounds for hydrogen evolution. Thermal pre-treatment of commercial TiO_2_ Degussa P25 (DTiO_2_) and Hombifine N (HTiO_2_) in the range from 600 to 800 °C was carried out followed by the deposition of copper oxides (1–10 wt. % of Cu). The morphology and chemical state of synthesized photocatalysts were studied using X-ray diffraction, UV–Vis diffuse reflectance spectroscopy, high-resolution transmission electron microscopy, X-ray photoelectron spectroscopy, and XANES/EXAFS X-ray absorption spectroscopy. Photocatalytic activity was tested in the hydrogen evolution from aqueous solutions of glycerol under ultraviolet (λ = 381 nm) and visible (λ = 427 nm) light. The photocatalysts 2% CuO_x_/DTiO_2_ T750 and 5% CuO_x_/DTiO_2_ T700 showed the highest activity under UV irradiation (λ = 380 nm), with the rate of H_2_ evolution at the level of 2.5 mmol (H_2_) g^−1^ h^−1^. Under the visible light irradiation (λ = 427 nm), the highest activity of 0.6 mmol (H_2_) g^−1^ h^−1^ was achieved with the 5% CuO_x_/DTiO_2_ T700 photocatalyst. The activity of these photocatalysts is 50% higher than that of the platinized 1% Pt/DTiO_2_ sample. Thus, it was shown for the first time that a simple heat treatment of a commercial titanium dioxide in combination with a deposition of non-noble metal particles led to a significant increase in the activity of photocatalysts and made it possible to obtain materials that were active in hydrogen production under visible light irradiation.

## 1. Introduction

The use of hydrogen as an energy source makes it possible to solve many problems associated with environmental pollution. Currently, there are various industrial methods for producing hydrogen, such as steam reforming of natural gas, coal gasification, biomass processing, etc. [1]. Unfortunately, as a rule, these methods are energy-consuming and can be cost-effective only for large-scale production. Therefore, one of the promising methods of hydrogen production is the use of renewable energy sources, in particular its photocatalytic synthesis under the action of solar radiation. The photocatalytic production of hydrogen is also of interest because hydrogen can be produced from inexpensive raw materials: water and many organic compounds, e.g., ethanol, methanol, glycerol, sugars, or carboxylic acids [2].

The photocatalytic splitting of water using semiconductors as a photocatalyst was first described in 1972 [3], and this initiated the worldwide development of photocatalytic hydrogen production. However, photocatalytic water splitting is accompanied by the recombination of photogenerated charge carriers, which reduces the quantum efficiency of the process. An addition of various compounds as electron donors can hinder the recombination of electron–hole pairs formed in the photocatalytic process and makes it possible to obtain hydrogen without admixture of oxygen [4,5]. In addition, the use of organic compounds as the sacrificial agent is attractive from a practical point of view. Indeed, along with hydrogen production, it helps to solve important environmental problems associated with water purification from various organic pollutants [6,7,8,9]. Among the various organic substances used to produce hydrogen, glycerol is of particular interest [10]. Indeed, since glycerol is a by-product of the synthesis of biodiesel from vegetable oils, the growing production of biodiesel will induce an increase in the production of glycerol as well. Thus, the use of glycerol as an electron donor in photocatalytic systems can help reduce the cost of hydrogen production [11,12].

The most common photocatalysts are semiconductors capable of absorbing light in the ultraviolet and visible regions. There are a number of different materials that can be used as the basis for photocatalysts for hydrogen production, and one of them is titanium dioxide [13,14,15,16,17]. This compound attracts special attention due to its stability under the action of light, availability, nontoxicity, and relatively low cost [5]. The band gap of TiO_2_ is 3.2 eV for the anatase phase and 3.0 eV for the rutile phase; therefore, TiO_2_ is photocatalytically active only under UV irradiation. Since UV radiation in sunlight constitutes only a small part (about 5%), it is necessary to increase the activity of TiO_2_ under visible light [18]. A common method here is to reduce the band gap. It can be achieved via several approaches, for example, by calcination of TiO_2_ at high temperatures and doping it with metals [19,20,21,22,23,24]. As a dopant, copper has attracted attention due to its high electron work function, which greatly improves the separation efficiency of photogenerated electrons and holes [25,26,27]. Copper oxides are promising cocatalysts in the processes under visible light irradiation, since both CuO and Cu_2_O have a narrow band gap and absorb light in the visible region [27,28].

The purpose of this work was to develop a method for the synthesis of photocatalysts based on TiO_2_ and copper compounds for the photocatalytic production of hydrogen under UV and visible irradiation. Photocatalysts with the composition of CuOx/TiO_2_ (Degussa P25) and CuOx/TiO_2_ (Hombifine N) were synthesized by the deposition of a cocatalyst on the surface of TiO_2_, which was preliminarily calcined at different temperatures. The catalysts were tested in the photocatalytic production of hydrogen from aqueous solutions of glycerol. The effects of calcination temperature and cocatalyst deposition on the activity in the target process were determined.

## 2. Materials and Methods

### 2.1. Reagents

The CuOx/TiO_2_ catalysts were synthesized using the following reagents: Cu(NO_3_)_2_·3H_2_O (Acros Organics, 98%+, Geel, Belgium), H_2_PtCl_6_·6H_2_O (Aurat, Pt 37.8%+, Moscow, Russia), NaBH_4_ (Acros Organics, 98%+, Geel, Belgium), Na_2_S (Acros Organics, 60%+, Geel, Belgium), TiO_2_ Degussa (Evonik) P25 (Evonik Industries, Essen, Germany), and TiO_2_ Hombifine N (Sachtleben Chemie, Duisburg, Germany). Photocatalytic experiments were carried out using analytical-grade glycerol (Reakhim, Moscow, Russia).

### 2.2. Pretreatment of TiO_2_ Samples

The TiO_2_ samples were obtained using titanium dioxides of commercial grades, Degussa P25 and Hombifine N. For calcination, 500 mg of TiO_2_ was placed in a crucible and kept in a muffle furnace at a constant temperature (600–850 °C in increments of 50 °C) for 3 h.

### 2.3. Synthesis of Photocatalysts CuO_x_/TiO_2_ and Pt/TiO_2_

To obtain the photocatalysts with 1, 2, 5, and 10 wt. % of copper, TiO_2_ samples (495, 490, 475, and 450 mg, respectively) were impregnated with a proper amount of 0.1 M solution of Cu(NO_3_)_2_ under constant stirring for 40 min, and then an excess of a fresh 0.1 mol solution of NaBH_4_ was added and stirred for 1 h. The resulting suspension was washed and centrifuged 5 times and then dried in air at 50 °C for 5 h. To prepare a 1 wt. % Pt/TiO_2_ sample, a TiO_2_ sample (495 mg) was impregnated with a 0.2 M solution of H_2_PtCl_6_ and then reduced with NaBH_4_ and then washed and dried in the same way as for photocatalysts with deposited copper.

In the case of using Hombifine N, the synthesized samples contained 1 wt. % and 5 wt. % Cu. In the case of using Degussa P25, the samples contained 1, 2, and 5 wt. % Cu. The obtained photocatalysts were labeled as y% CuOx/DTiO_2_ T or y% CuOx/HTiO_2_ T, where y corresponds to the weight content of copper, and T is the temperature of TiO_2_ calcination, DTiO_2_ denotes TiO_2_ Degussa P25, and HTiO_2_ denotes TiO_2_ Hombifine N. For example, the label 5% CuOx/DTiO_2_ T700 denotes a TiO_2_ Degussa P25 sample calcined at 700 °C and modified with copper with a calculated content of 5 wt. %. We decided to indicate the mass percentage of copper, because it was difficult to determine the stoichiometric composition of CuOx and accordingly calculate the content of copper oxide.

### 2.4. Sample Testing Methods

#### 2.4.1. Physical Methods

The synthesized samples were characterized by X-ray diffraction (XRD), low-temperature nitrogen adsorption, diffuse reflectance spectroscopy (DRS), X-ray photoelectron spectroscopy (XPS), high-resolution transmission electron microscopy (HR TEM), and XANES/EXAFS X-ray absorption spectroscopy.

The phase composition of the photocatalysts was determined by XRD with a Bruker D8 Advance diffractometer (Bruker AXS GmbH, Ettlingen, Germany) using monochromatized Cu Kα radiation with a wavelength of 1.5418 Å. The crystal size (CS) was estimated as the coherent scattering domain size using the Scherrer formula. The specific surface area (SSA) and pore volume of the catalysts were obtained by low-temperature N_2_ adsorption–desorption (N_2_ adsorption at 77 K) using an ASAP 2400 apparatus (ASAP Industries Manufacturing, Houma, LA, USA). Their optical properties were studied by the DRS method. Diffuse reflection spectra in UV and visible regions were obtained using a Shimadzu UV-2501 PC spectrophotometer (Shimadzu, Kyoto, Japan) with an ISR-240A diffuse reflection attachment.

The chemical composition of the catalyst surface was studied by XPS with a photoelectron spectrometer (SPECS Surface Nano Analysis GmbH, Berlin, Germany) using non-monochromatized Al Kα radiation (hυ = 1486.6 eV). The spectrometer was equipped with a PHOIBOS-150 hemispherical analyzer (SPECS Surface Nano Analysis GmbH, Berlin, Germany) and an XR-50 X-ray source with a double Al/Mg anode. The charging effect was corrected using the binding energy of the Ti2p3/2 peak at 459.0 eV.

The chemical state of copper in the bulk of the catalysts was studied using XANES X-ray absorption spectroscopy at the station of the Kurchatov Synchrotron Radiation Source (Moscow, Russia). The electron energy in the storage ring was 2.5 GeV at a beam current of 50–150 mA. To monochromatize synchrotron radiation, we used a silicon single crystal with (111) orientation in the form of a cut-out monoblock mounted on a goniometric head. The energy resolution achieved was ΔE/E = 2 × 10−4. The X-ray absorption spectra of the catalysts were obtained in fluorescence geometry (a sample with 20% Cu was tested in the transmission mode). The X-ray beam intensity before and after passing through the sample was measured using ionization chambers equipped with Keithley 6487 digital picoammeters.

The microstructure of the photocatalysts was studied by HRTEM using a ThemisZ electron microscope (Thermo Fisher Scientific, Waltham, MA, USA) at an accelerating voltage of 200 kV. The microscope was equipped with a SuperX energy-dispersive spectrometer and a spherical aberration corrector. The maximum resolution of the microscope was 0.06 nm. For the HR TEM analysis, the samples were ultrasonically dispersed onto perforated carbon substrates attached to aluminum grids.

#### 2.4.2. Catalytic Tests

**Figure 1:**
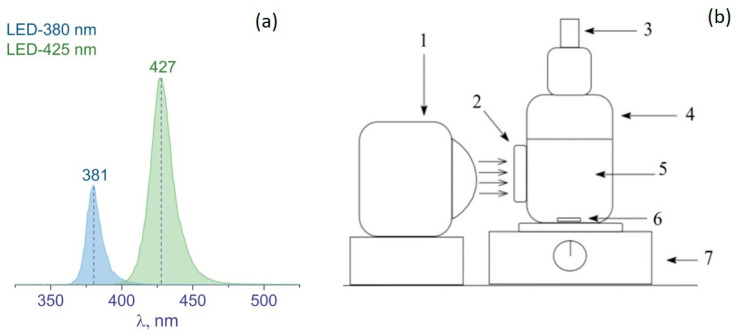
(**a**) Spectra of LEDs with the wavelength at maximum intensity; (**b**) scheme of reaction set-up for photocatalytic study. 1—LED, 2—quartz window, 3—sampler, 4—reactor, 5—reaction mixture, 6—stir bar, 7—magnetic stirrer.

The photocatalytic activity of synthesized samples was determined using the setup shown in Figure 1. The setup consisted of a glass reactor with a quartz window (S_window_ = 22 cm^2^), an LED source of irradiation, and a magnetic stirrer.

The reaction mixture consisted of a photocatalyst (50 mg) and a 2.8% aqueous solution of glycerol. The total volume of the suspension was 100 mL. Before the experiment, the reaction mixture was purged with Ar for 15 min to remove atmospheric oxygen. After the purge, either an LED-381 nm (for UV irradiation) or an LED-427 nm (for visible light irradiation) was turned on (Figure 1). Under irradiation of the photocatalyst, the reaction mixture evolved hydrogen. During the experiment, the gas phase (250 μL) was sampled with a gas syringe (Hamilton) every 15 min. The experiment lasted from 90 to 150 min, depending on the photocatalyst activity. The amount of hydrogen was determined using a Chromos GC-1000 chromatograph (Chromos, Moscow, Russia) with a thermal conductivity detector and with Ar as the carrier gas.

## 3. Results and Discussion

### 3.1. Physical Methods

#### 3.1.1. XRD and BET Methods

**Figure 2:**
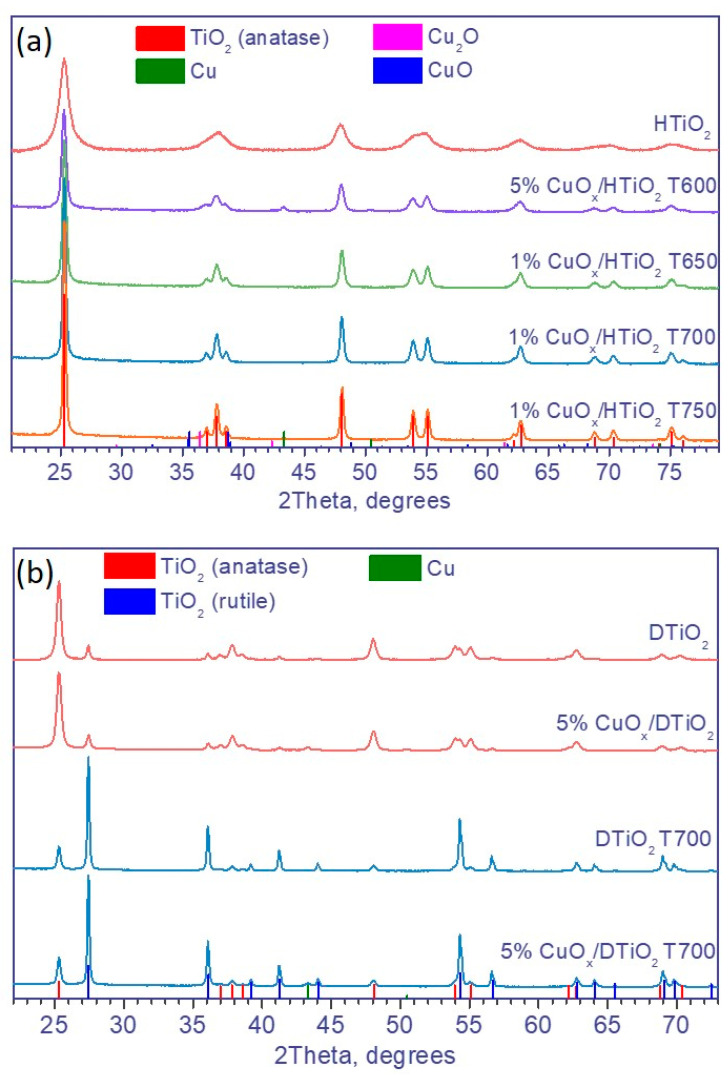
XRD patterns of the samples based on (**a**) TiO_2_ Hombifine N and (**b**) TiO_2_ Degussa P25.

The changes in the structure of TiO_2_ during its calcination were studied by XRD. The X-ray diffraction patterns of the photocatalysts CuOx/HTiO_2_ with copper contents of 1 and 5 wt. % calcined at temperatures from 600 to 750 °C are shown in Figure 2a. As can be seen, the patterns show only peaks due to the anatase phase with the addition of low-intensity peaks due to copper and copper oxides. Since there are no rutile peaks, it can be concluded that Hombifine N does not undergo any phase transformation at these calcination temperatures.

In contrast, the calcination of Degussa P25 leads to the phase transition of anatase to rutile. As seen from Table 1 and Figure 2b, the calcination of Degussa P25 at temperatures from 600 to 750 °C leads to an increase in the rutile content, and starting from 800 °C, the sample contains only the rutile phase. The textural characteristics of the Degussa P25 samples were determined using low-temperature nitrogen adsorption. As seen from Table 1, the specific surface area and pore volume of the uncalcined TiO_2_ sample and the sample calcined at 600 °C practically coincide, whereas the calcination at 700 °C or 800 °C leads to a significant decrease in the specific surface area and pore volume. Thus, the calcination of the commercial titanium dioxides at temperatures up to 600 °C does not change the textural characteristics and phase composition of the samples.

The XRD data imply that the thermal treatment of the photocatalysts based on Hombifine N could not lead to an increase in their photocatalytic activity (see below). The samples based on Degussa P25 were characterized in more detail.

#### 3.1.2. UV–Vis Spectroscopy

**Figure 3:**
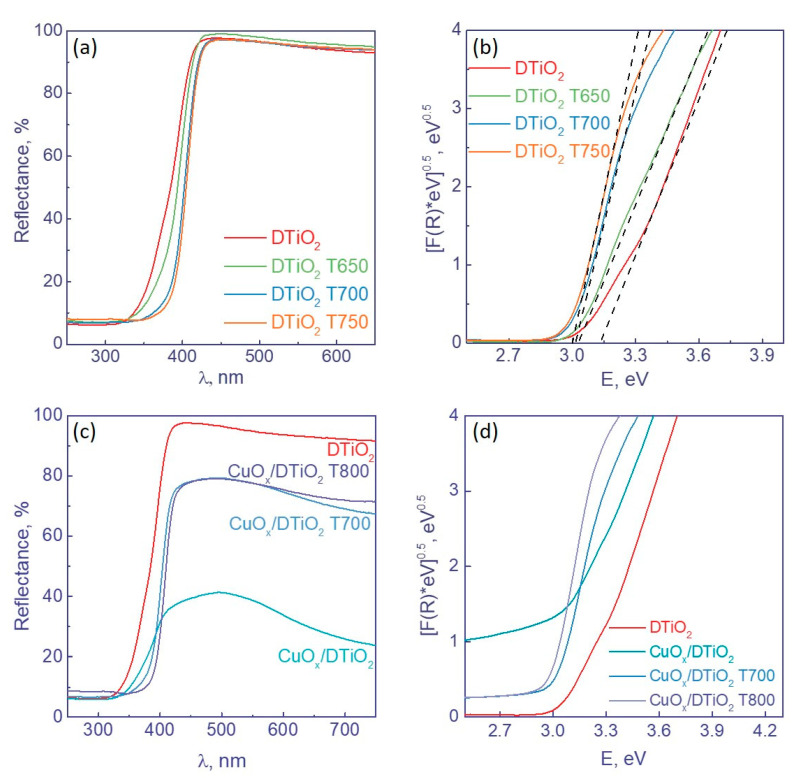
(**a**,**c**) Diffuse reflectance spectra of photocatalysts and (**b**,**d**) Tauc plots with tangent to graph (dotted line).

The optical properties of uncalcined Degussa P25 and Degussa P25 calcined at temperatures from 650 to 750 °C were studied by DRS. It is found that the calcination of this TiO_2_ shifts the absorption edge to the longer waves due to an increase in the content of rutile (Figure 3a), which is consistent with the XRD data.

To determine the band gap, the absorption spectra were plotted in the Tauc coordinates (Figure 3b). First, the adsorption coefficient F(R) was found from the DRS data according to the Kubelka–Munk Equation (1):

(1) $$F\left(R\right)=\frac{{\left(1-R\right)}^{2}}{2R},$$

where *R* is the reflection coefficient of the sample. Then, the absorption curves were plotted in the Tauc coordinates for allowed indirect transitions. According to the calculated data, the calcination leads to a decrease in the band gap from 3.14 eV (for the uncalcined sample) to ca. 3.0 eV for the samples calcined at temperatures from 650 to 750 °C.

In addition, the DRS method was used to study the samples with deposited cocatalysts. As seen from Figure 3c, the addition of cocatalysts also enhances the absorption in the visible region. In the case of Cu-containing catalysts, this effect is a result of the formation of copper oxides [29] (see the XPS and XANES results below).

#### 3.1.3. XPS and XANES Methods

**Figure 4:**
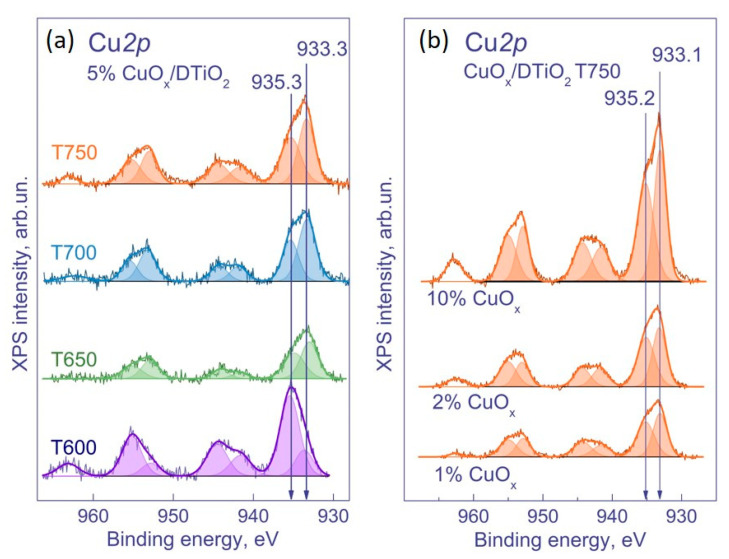
XPS spectra of Cu2*p* (**a**) 5% CuO_x_/DTiO_2_ T600–T750; (**b**) (1–10)% CuO_x_/DTiO_2_ T750.

The surface of the samples CuO_x_/DTiO_2_ was characterized using the XPS method (Figure 4). There were two series of the samples: a series 5% CuO_x_/DTiO_2_ *T* (where *T* = 600, 650, 700, and 750 °C) and a series *y* % CuO_x_/DTiO_2_ T750 (where *y* = 1, 2, 5, and 10 wt. %). With their use, we studied how the mass fraction of the copper cocatalyst and the temperature of the Degussa P25 pre-calcination affect the properties of the photocatalyst.

The Cu*2p*_3/2_ spectra of the catalysts show two symmetric peaks at 933.0–933.6 eV and 934.8–935.4 eV, respectively. The spectra also contain two shake-up satellites, which are related to the peak at 934.8–935.4 eV. These data indicate that the peak at 934.8–935.4 eV and its satellites belong to copper in the Cu^2+^ state (presumably in the composition of CuO), while the peak at 933.0–933.6 eV corresponds to copper in the state Cu^0^ or Cu^1+^. It should be noted that the Cu*2p*_3/2_ binding energies of the Cu^1+^ and Cu^0^ states are close; thus, the unambiguous identification of these states using XPS is complicated. However, as seen from Table 2, the predominant state of copper in the samples is Cu^2+^.

**Figure 5:**
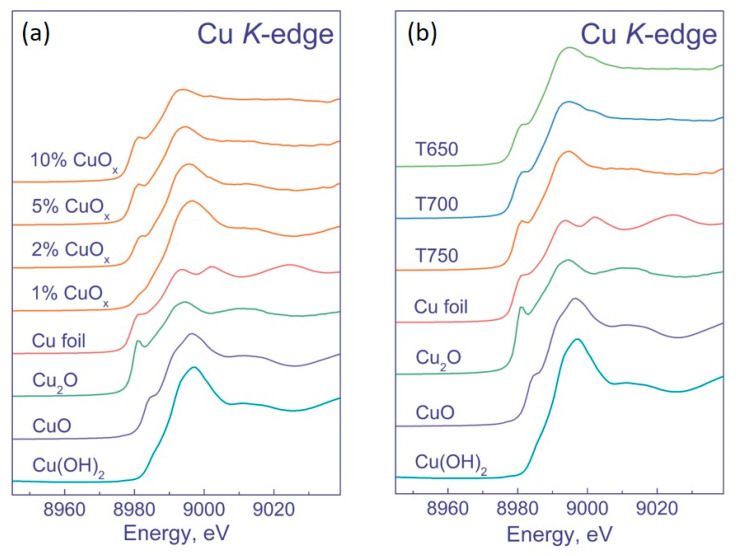
XANES spectra of the K-absorption edge of Cu (**a**) (1–10)% CuO_x_/DTiO_2_ T750; (**b**) 5% CuO_x_/DTiO_2_ T650–T750 in comparison with the spectra of metallic copper, Cu_2_O, CuO, and Cu(OH)_2_.

Unfortunately, the XPS method does not provide unambiguous information on the state of copper; moreover, under conditions of an XPS experiment, Cu^2+^ can be reduced to Cu^1+^ under X-ray radiation. Therefore, to clarify the state of copper in the samples, they were studied by XANES (Figure 5). We tested the samples prepared based on DTiO_2_ pretreatment at 750 °C. As a result, it was shown that Cu^2+^ in the samples was in the form of Cu(OH)_2_ and CuO. With an increase in the amount of copper, the percentage of Cu^2+^ decreases, and the percentage of Cu^1+^ in the form of Cu_2_O increases (Table 3). In addition, an increase in the amount of copper leads to the appearance of copper in the metallic state. Its amount gradually increases and achieves the maximum in the sample 5% CuO_x_/DTiO_2_ T700.

At the Cu *K*-edge, the sample 1% Cu/DTiO_2_ T750 has a broad intense peak in the region of 8996.2 eV, which is characteristic of copper in the Cu^2+^ state. This state can exist in the form of Cu(OH)_2_, Cu(NO_3_)_2_, and CuO nanoparticles; thus, a comparison with the reference spectra in this case does not allow an unambiguous conclusion about the state of copper in this sample [30,31,32]. The spectra of other samples show a shoulder at the absorption edge in the region of 8981.2–8981.9 eV, which is characteristic of bulk CuO, Cu_2_O, and metallic copper. With an increase in the amount of deposited copper, its oxidation state decreases from Cu^2+^ to Cu^0^.

**Figure 6:**
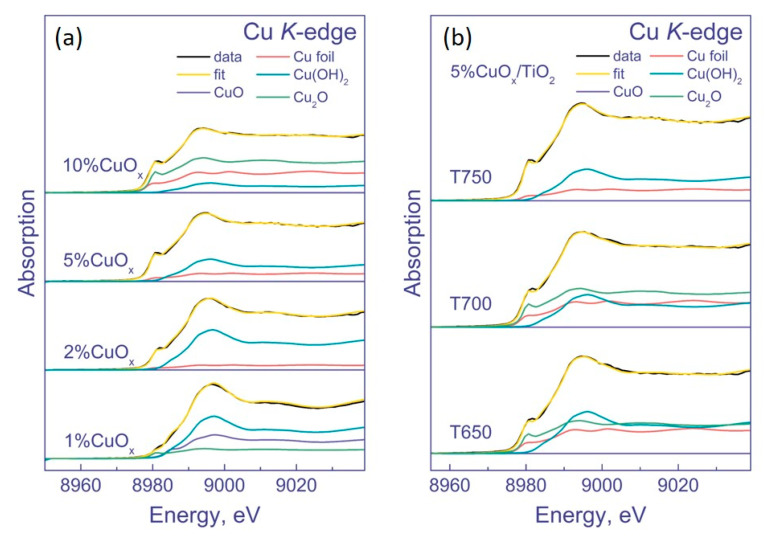
XANES spectra of the K-edge of Cu and their decomposition into a linear set of spectra of metallic copper, CuO, Cu_2_O, and Cu(OH)_2_ (**a**) (1–10)% CuO_x_/DTiO_2_ T750; (**b**) 5% CuO_x_/DTiO_2_ T650–T750.

The spectra of the 5 wt. % CuO_x_/DTiO_2_ samples obtained at different calcination temperatures are similar. However, upon closer examination, it can be noted that the increase in the calcination temperature from 650 to 750 °C leads to the sharpening of both the peak and the shoulder. A sharper shoulder at the absorption edge is characteristic of copper in the form of Cu_2_O, and its appearance indicates that higher calcination temperatures facilitate the formation of this oxide. To clarify the chemical composition of the samples, their Cu *K*-edge XANES spectra were fitted by a linear combination of reference spectra in the energy range from −20 to 30 eV relative to the absorption edge (Figure 6).

Table 3 presents the results of the linear combination fitting (LCF). As seen, the sample with 1% Cu contains approximately half of the copper in the Cu^2+^ state in the form of Cu(OH)_2_, while the rest of the copper is in the form of CuO and Cu_2_O. As the amount of copper in the samples increases, the percentage of Cu^2+^ decreases and Cu^1+^ increases, so that for the samples 5–10% CuO_x_/DTiO_2_ T750, the percentage of Cu^1+^ achieves 50–60%. The percentage of copper in the metallic state also increases and achieves the maximum in the sample 10% CuO_x_/DTiO_2_ T750. Analyzing the state of copper in the 5 wt. % Cu sample, one can see that an increase in calcination temperature leads to an increase in the percentage of Cu^1+^ and a decrease in the percentage of Cu^2+^ and metallic Cu.

#### 3.1.4. HR TEM Method

**Figure 7:**
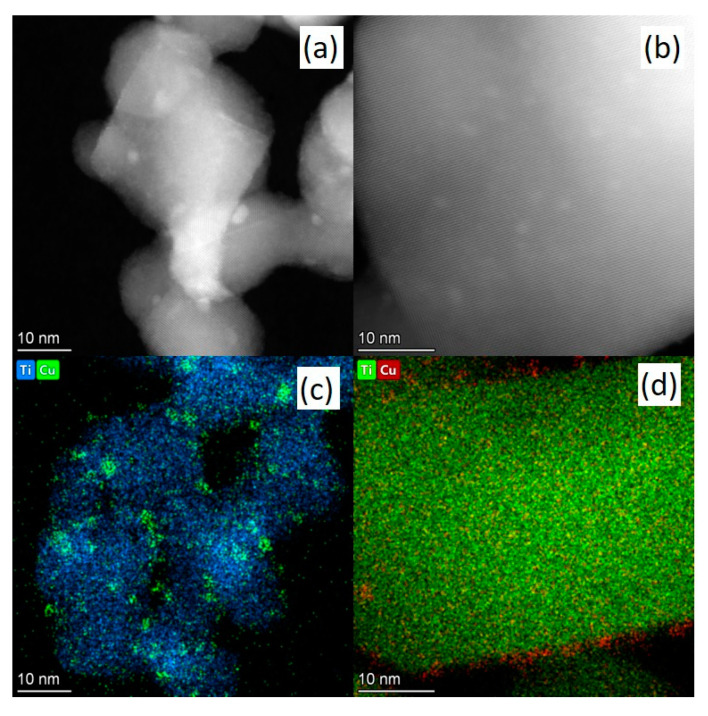
HAADF STEM micrographs of (**a**) CuO_x_/DTiO_2_ and (**b**) CuO_x_/DTiO_2_ T700; EDX mapping of (**c**) CuO_x_/DTiO_2_ and (**d**) CuO_x_/DTiO_2_ T700.

The samples 5 wt. % CuO_x_/DTiO_2_ (without calcination) and 5 wt. % CuO_x_/DTiO_2_ T700 were studied by HRTEM in the dark field mode (Figure 6), which provides high contrast images with the bright regions corresponding to heavier atoms. As seen from Figure 7, the particle size of copper in the sample 5 wt. % CuO_x_/DTiO_2_ is in a wide range from 1.5 to 5 nm, whereas the deposition of copper on calcined TiO_2_ facilitates the formation of Cu particles with a size of 1–2 nm. It can also be seen (Figure 7c) that in the sample 5 wt. % CuO_x_/DTiO_2_, copper forms surface agglomerates, whereas in the sample 5 wt. % CuO_x_/DTiO_2_ T700, it is uniformly distributed over the surface (Figure 7d). Probably, the uniform distribution of copper is a result of the following effect: the calcination of DTiO_2_ leads to the formation of various defects that act as fixation centers for copper particles, thus preventing their agglomeration. Figure S1 (scale 1 μm) shows that the calcination at 700 C leads to a growth of titanium dioxide particles, which completely confirms the XRD data.

Thus, the entire set of physicochemical methods demonstrates that the preliminary calcination of DTiO_2_ significantly affects not only its structural and textural properties but also the state of the cocatalyst: the calcination of DTiO_2_ at temperatures above 600 °C leads to a shift of the absorption edge to longer wavelengths and provides the uniform deposition of the cocatalyst on the DTiO_2_ surface.

### 3.2. Kinetic Experiments

#### 3.2.1. Activity of Photocatalysts under UV Irradiation

**Figure 8:**
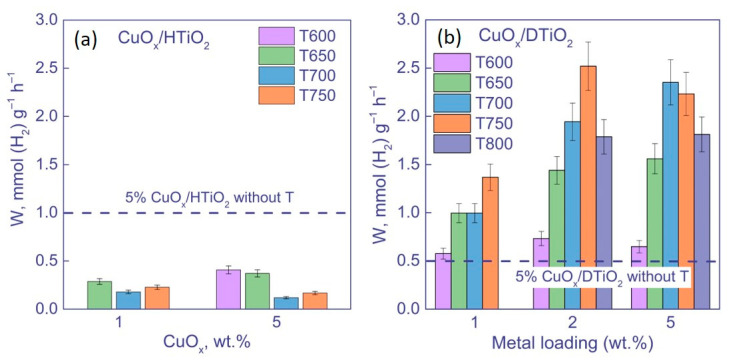
Rates of H_2_ evolution over (**a**) CuO_x_/HTiO_2_ and (**b**) CuO_x_/DTiO_2_. Conditions: m(cat.) = 50 mg, V = 100 mL, C_0_(glycerol) = 0.38 M, λ = 381 nm.

In these experiments, we studied how the kinetics and rate of photocatalytic hydrogen evolution depend on the amount of cocatalyst and the temperature of TiO_2_ calcination (Figure 8).

As seen, in the series of HTiO_2_ samples, the photocatalyst with 5 wt. % CuO_x_ on uncalcined TiO_2_ has the highest activity: the hydrogen evolution rate over it achieves 1.0 ± 0.1 mmol (H_2_) g^−1^ h^−1^ (Figure 8a). In contrast, the catalysts based on calcined HTiO_2_ show much lower activity (about 0.2–0.4 mmol (H_2_) g^−1^ h^−1^). As noted above, the calcination of Hombifine N does not lead to the conversion of anatase to rutile, and as a result, the photocatalytic activity of the calcined samples is lower due to a decrease in their specific surface area and increase in crystallite size. The higher activity of the samples with the higher content of cocatalyst is caused by an increase in the efficiency of charge separation due to the transfer of photogenerated electrons between TiO_2_ and copper compounds.

For the series of CuO_x_/DTiO_2_ samples, the rates of hydrogen formation are significantly higher than in the previous case. At that, the reaction rate increases with an increase in the calcination temperature (Figure 8b). In contrast to the previous series, the calcination of TiO_2_ at temperatures of 700–750 °C provides a significant increase in the activity of photocatalysts, while the further increase in the calcination temperature to 800 °C leads to the formation of less active photocatalysts. Indeed, the rate of hydrogen evolution over the uncalcined sample is 0.5 mmol (H_2_) g^−1^ h^−1^, whereas the most active samples (2 wt. % CuO_x_/DTiO_2_ T750 and 5 wt. % CuO_x_/DTiO_2_ T700) provide the rates of 2.5 and 2.4 mmol (H_2_) g^−1^ h^−1^, respectively. It should be noted that these rates, obtained under UV irradiation, are among the highest published for TiO_2_-based systems (Table 4). As shown in Section 3.1.3, the calcination of DTiO_2_ leads to an increase in the amount of rutile and to a decrease in the sample surface area. Thus, based on the results of kinetic experiments, it can be concluded that, for the photocatalytic production of H_2_, a decrease in the band gap of the photocatalyst is of crucial importance.

#### 3.2.2. Activity of Photocatalysts under Visible Light Irradiation

**Figure 9:**
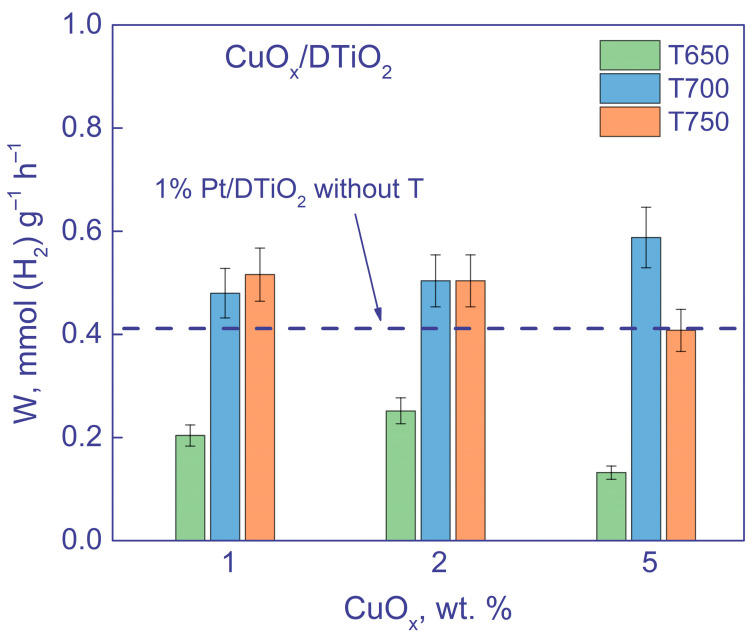
Rates of H_2_ evolution over CuO_x_/DTiO_2_. Conditions: m(cat.) = 50 mg, V = 100 mL, C_0_(glycerol) = 0.38 M, λ = 427 nm.

As noted, a significant part of the solar spectrum is represented by visible light. Therefore, it was of interest to study the activity of the synthesized catalysts under visible light irradiation (Figure 9).

The activities of all the photocatalysts are summarized in Table 5. As seen, the photocatalysts based on HTiO_2_ are inactive under visible light. The activity of photocatalysts based on DTiO_2_, as in the case of UV irradiation, significantly increases after calcination. The maximum activity of 0.6 ± 0.06 mmol (H_2_) g^−1^ h^−1^ was achieved with the photocatalyst 5 wt. % CuO_x_/DTiO_2_ 700, as well as in the case of UV irradiation (Figure 8), while the activity of the uncalcined sample 1% CuO_x_/DTiO_2_ was close to zero, the same as for all samples calcined at 600. Note that the photocatalysts 5 wt. % CuO_x_/DTiO_2_ 700 demonstrate the activity that exceeds the activity of 1% Pt/DTiO_2_ by 50%. Moreover, the obtained activity exceeds that for similar systems described in the literature (Table 4).

Therefore, the highest activity both under UV irradiation and visible light is shown by the sample 5 wt. % CuO_x_/DTiO_2_ 700. This fact can be explained by the optimal combination of the following factors:

- An increase in the absorption of the photocatalyst due to the shift in the absorption edge of DTiO_2_ and the deposition of copper compounds as a cocatalyst;
- The optimal ratio of anatase and rutile phases for the formation of heterojunctions;
- The size of DTiO_2_ particles after calcination at 700 °C does not increase as much as after calcination at higher temperatures;
- Deposition of Cu/CuO_x_ on the surface of calcined DTiO_2_ leads to a uniform distribution of small cocatalyst particles;
- The presence of copper in the composition of various compounds (Cu, Cu_2_O, Cu(OH)_2_) facilitates the formation of a complex set of photogenerated charge heterojunctions, which significantly increases the electron lifetime and process efficiency.

Thus, when light reaches the photocatalyst, an electron–hole pair is generated both on titanium dioxide and on copper compounds. The presence of copper in various states significantly increases the absorption of light by the photocatalyst in the visible region [35]. Depending on the mutual arrangement of copper particles, copper oxides, and titanium dioxide, various types of heterojunctions can be formed [36]. If metallic copper is located between the particles of titanium dioxide and copper oxide, the charge transfer Z-scheme is realized, and proton reduction occurs on the particles of Cu_2_O. Electrons in the conduction band of Cu_2_O have a significant reduction potential, which greatly accelerates the rate of photocatalytic hydrogen production [37].

## 4. Conclusions

Thus, a series of photocatalysts based on TiO_2_ of commercial grades, Hombifine N (HTiO_2_) and Degussa P25 (DTiO_2_), modified with copper compounds such as Cu, Cu_2_O, CuO, and Cu(OH)_2_ was synthesized and characterized by a set of physicochemical methods. It has been shown that the calcination of DTiO_2_ at temperatures above 600 °C leads to a significant increase in the amount of the rutile phase in the photocatalyst, a shift in the absorption edge, and a uniform distribution of cocatalyst particles on the TiO_2_ surface. The calcination of Hombifine N does not lead to the transformation of anatase to rutile. It has been shown that an increase in the amount of rutile and the addition of copper in the Cu^2+^ oxidation state increase the absorption of DTiO_2_ photocatalysts in the visible region and accordingly provide their high activity under visible light irradiation.

The samples 2% CuO_x_/DTiO_2_ T750 and 5% CuO_x_/DTiO_2_ T700 showed the maximum activity under UV irradiation (380 nm) in obtaining hydrogen from aqueous solutions of glycerol, with the rate of H_2_ evolution at the level of 2.5 mmol (H_2_) g^−1^ h^−1^. Under the visible light irradiation (427 nm), the highest activity of 0.6 mmol (H_2_) g^−1^ h^−1^ was achieved with the 5% CuO_x_/DTiO_2_ T700 photocatalyst. The activity of these photocatalysts is 50% higher than that of the platinized 1% Pt/DTiO_2_ sample. The pre-calcination of Hombifine N does not lead to the enhancement of photocatalyst activity due to the absence of phase transformation. It has been shown that the presence of CuO increases the activity of the photocatalysts to the greatest extent, and the reduction of Cu^2+^ to the Cu^+^ or Cu^0^ states leads to a slowdown of the H_2_ evolution.

In general, it can be concluded that a simple heat treatment of a commercial titanium dioxide in combination with a deposition of non-noble metal particles (e.g., copper) leads to a significant increase in the activity of photocatalysts and makes it possible to obtain materials that are active in the formation of hydrogen under visible light irradiation.

## Figures and Tables

**Table 1:** Structural and textural characteristics of calcined Degussa P25 samples.

No.	Sample	Phase Composition, wt. %	CS, nm	SSA, m^2^ g^−1^	V, cm^3^ g^−1^
1	DTiO_2_	85% (Anatase)/15% (Rutile)	19/30	55	0.48
2	DTiO_2_ 600	75% (Anatase)/25% (Rutile)	21/40	55	0.52
3	DTiO_2_ 650	68% (Anatase)/32% (Rutile)	35/65	41	0.16
4	DTiO_2_ 700	36% (Anatase)/63% (Rutile)	45/85	19	0.074
5	DTiO_2_ 750	5% (Anatase)/95% (Rutile)	62/>100	12	0.046
6	DTiO_2_ 800	100% (Rutile)	>100	10	0.035

**Table 2:** Surface contents of atoms for the analyzed photocatalysts according to XPS data.

No.	Sample	[Cu]/[Ti]	[O]/[Ti]	Cu^2+^, %
1	5% CuO_x_/DTiO_2_ T600	0.26	4.8	85
2	5% CuO_x_/DTiO_2_ T650	0.14	3.3	52
3	5% CuO_x_/DTiO_2_ T700	0.22	3.2	51
4	5% CuO_x_/DTiO_2_ T750	0.24	3.4	60
5	1% CuO_x_/DTiO_2_ T750	0.17	3.1	63
6	2% CuO_x_/DTiO_2_ T750	0.25	3.2	64
7	10% CuO_x_/DTiO_2_ T750	0.48	3.7	64

**Table 3:** Phase composition of the samples according to the results of the linear combination fitting of Cu *K*-edge XANES spectra.

No.	Sample	Cu^2+^, %	Cu^1+^, %	Cu^0^, %
Weight content of copper				
1	1% CuO_x_/DTiO_2_ T750	85	15	–
2	2% CuO_x_/DTiO_2_ T750	49	42	8
3	5% CuO_x_/DTiO_2_ T750	27	59	13
4	10% CuO_x_/DTiO_2_ T750	12	54	35
Temperature of TiO_2_ Degussa P25 calcination				
1	5% CuO_x_/DTiO_2_ T650	36	36	28
2	5% CuO_x_/DTiO_2_ T700	28	42	30
3	5% CuO_x_/DTiO_2_ T750	27	59	13

**Table 4:** Activity of photocatalysts based on TiO_2_ in the formation of H_2_ from aqueous solutions of alcohols.

Photocatalyst	Modification Method	Electron Donor	Radiation Source	W, μmol (H_2_) g^−1^ h^−1^	AQE, %	Ref
Fe/TiO_2_	Doping with metal atoms and deposition of metals	10% Ethanol	Xenon lamp, 500 W (λ > 400 nm)	248	0.1	[22]
Ni/TiO_2_				205		
Ag/TiO_2_				265		
Fe-Ni/TiO_2_				348		
Fe/Ag/TiO_2_				512		
Ni/Ag/TiO_2_				336		
Fe-Ni/Ag/TiO_2_				793	0.2	
Cr-N@TiO_2_	Doping with non-metal atoms and metal cations	10% Glycerol	Medium pressure mercury lamp, 450 W	126	-	[24]
Co-N@TiO_2_				313		
Ni-N@TiO_2_				506		
Cu-N@TiO_2_				1615		
1.5% Au@TiO_2_ (nanotubes)	Vacuum-assisted-impregnation route	25% Methanol	Xenon lamp, 100 mW·cm^−1^ (λ > 400 nm)	482	0.1	[33]
1.5% Au/TiO_2_ (nanotubes)				223		
CuO_x_/TiO_2_	Surface deposition of metals	0.5% Glycerol	LED source, 30 W (λ = 380 nm)	550	-	[12]
Pt/TiO_2_				1350		
80% Cu_2_O/TiO_2_	Microemulsion method	20% Methanol	Xenon lamp	215	-	[34]
60% Cu_2_O/TiO_2_				892		
40% Cu_2_O/TiO_2_				1242		
30% Cu_2_O/TiO_2_				1388		
20% Cu_2_O/TiO_2_				1345		
TiO_2_				28		
Cu_2_O				3		
2% Cu/TiO_2_	Impregnation reduction	25% Methanol	UV lamp, 300 W (λ = 340 nm)	5000	-	[35]
2% Ni/TiO_2_				2300		
2% Co/TiO_2_				2250		
2% Zn/TiO_2_				200		
2% Cu/TiO_2_			Halogen lamp, 500 W (λ > 420 nm)	220	-	
2% Ni/TiO_2_				10		
2% Co/TiO_2_				20		
2% Zn/TiO_2_				11		

**Table 5:** Activity of synthesized photocatalysts.

No.	Sample	Catalytic Activity, mmol (H_2_) g^−1^ h^−1^	
		381 nm	427 nm
Cu/TiO_2_ (Degussa P25)			
1	1% CuO_x_/DTiO_2_	0.5 ± 0.05	0
2	1% CuO_x_/DTiO_2_ T600	0.6 ± 0.06	-
3	1% CuO_x_/DTiO_2_ T650	1.0 ± 0.1	0.2 ± 0.02
4	1% CuO_x_/DTiO_2_ T700	1.0 ± 0.1	0.5 ± 0.05
5	1% CuO_x_/DTiO_2_ T750	1.3 ± 0.1	0.5 ± 0.05
6	2% CuO_x_/DTiO_2_ T600	0.7 ± 0.07	—
7	2% CuO_x_/DTiO_2_ T650	1.4 ± 0.1	0.3 ± 0.03
8	2% CuO_x_/DTiO_2_ T700	2.0 ± 0.1	0.5 ± 0.05
9	2% CuO_x_/DTiO_2_ T750	2.5 ± 0.25	0.5 ± 0.05
10	5% CuO_x_/DTiO_2_ T600	0.6 ± 0.06	—
11	5% CuO_x_/DTiO_2_ T650	1.6 ± 0.2	0.1 ± 0.01
12	5% CuO_x_/DTiO_2_ T700	2.4 ± 0,2	0.6 ± 0.06
13	5% CuO_x_/DTiO_2_ T750	2.2 ± 0.2	0.4 ± 0.04
14	10% CuO_x_/DTiO_2_ T700	1.4 ± 0.1	—
15	10% CuO_x_/DTiO_2_ T750	1.0 ± 0.1	—
Cu/TiO_2_ (Hombifine N)			
16	5% CuO_x_/HTiO_2_ T600	0.4 ± 0.04	—
17	5% CuO_x_/HTiO_2_ T650	0.4 ± 0.04	—
18	5% CuO_x_/HTiO_2_ T700	0.1 ± 0.01	—
19	5% CuO_x_/HTiO_2_ T750	0.2 ± 0.02	—
20	1% CuO_x_/HTiO_2_ T650	0.3 ± 0.03	—
21	1% CuO_x_/HTiO_2_ T700	0.2 ± 0.02	—
22	1% CuO_x_/HTiO_2_ T750	0.2 ± 0.02	—

## Data Availability

The data presented in this study are available on request from the corresponding author.

## Supplementary Materials

The following supporting information can be downloaded at: https://www.mdpi.com/article/10.3390/nano12183106/s1, Figure S1: HAADF STEM micrographs of (a) CuO_x_/DTiO_2_ and (b) CuO_x_/DTiO_2_ T700.
