# Supplementary material for: Copper-Modified Titania-Based Photocatalysts for the Efficient Hydrogen Production under UV and Visible Light from Aqueous Solutions of Glycerol
Source: Nanomaterials (Basel). 2022 Sep 7;12(18):3106. doi: 10.3390/nano12183106 (PMC9504562; doi:10.3390/nano12183106)
Supplement: Supplementary file 1 [file nanomaterials-12-03106-s001.zip › nanomaterials-1883232-supplementary.pdf]

Supplementary Materials

Copper-Modified Titania-Based Photocatalysts for the Efficient Hydrogen Production under UV and Visible Light from Aqueous Solutions of Glycerol

Anna Yu. Kurenkova, Anastasiya Yu. Yakovleva, Andrey A. Saraev, Evgeny Yu. Gerasimov, Ekaterina A. Kozlova * and Vasily V. Kaichev

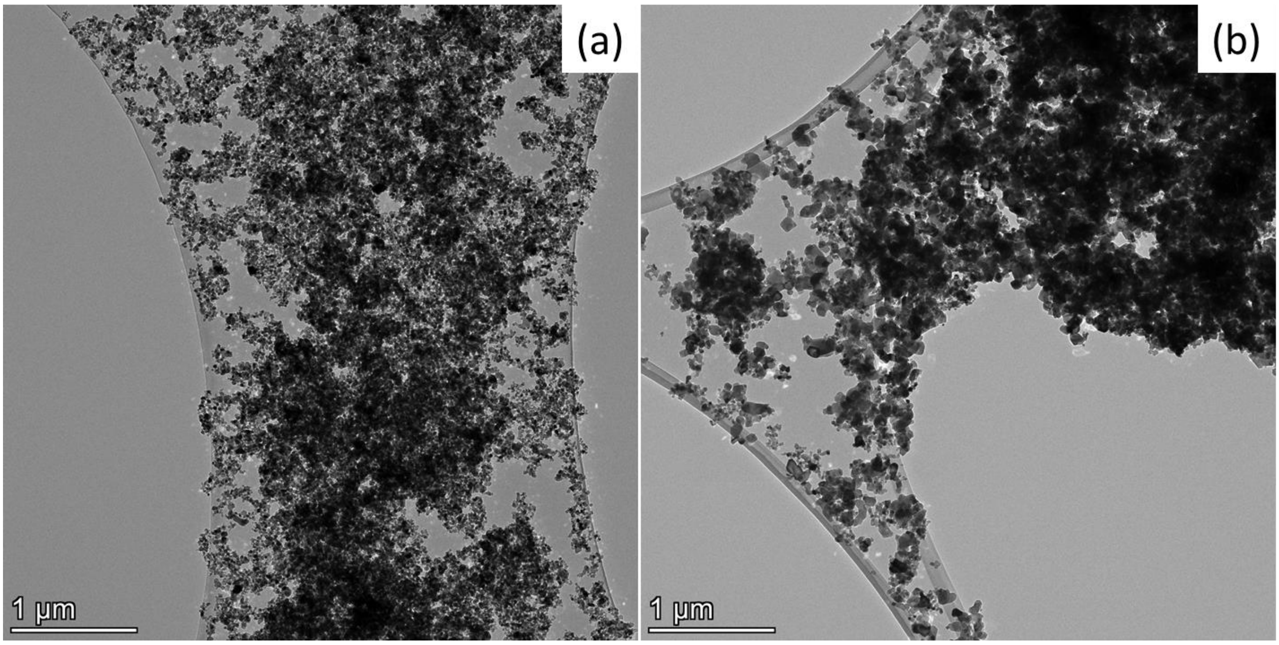

Figure S1. HAADF STEM micrographs of (**a**) $CuO_x/DTiO_2$ and (**b**) $CuO_x/DTiO_2$ T700
